# Epidemiology and classification for canine and feline mammary gland tumors: a histopathological survey of 437 mammary gland tumor biopsies performed in a secondary care hospital in Chiang Mai, Thailand from 2012 to 2019

**DOI:** 10.7717/peerj.17077

**Published:** 2024-03-15

**Authors:** Wanwisa Srisawat, Kidsadagon Pringproa, Worapat Prachasilchai, Atigan Thongtharb, Nattawooti Sthitmatee

**Affiliations:** 1 Graduate School of Veterinary Science, Faculty of Veterinary Medicine, Chiang Mai University, Muang, Chiang Mai, Thailand; 2 Laboratory of Veterinary Vaccine and Biological Products, Faculty of Veterinary Medicine, Chiang Mai University, Muang, Chiang Mai, Thailand; 3 Faculty of Veterinary Medicine, Chiang Mai University, Muang, Chiang Mai, Thailand

**Keywords:** Mammary gland tumors, Dog, Cat, Risk factor, Histopathological survey, Epidemiology

## Abstract

**Background:**

Metastatic disease resulting from mammary gland tumors (MGTs) is a known cause of death among dogs and cats. Keys to successful prevention and management strategies involve the accurate recording of diagnostic data.

**Methods:**

This retrospective study reviewed the epidemiology and classification of canine mammary gland tumors (CMTs) and feline mammary gland tumors (FMTs), as well as the factors including sex, age, and breed related to the occurrence of these tumors. Accordingly, 1,736 tumor biopsy cases were reported from 2012 to 2019 at Chiang Mai University Small Animal Hospital, Thailand, with 1,639 canine tumor biopsy cases and 97 feline tumor biopsy cases.

**Results:**

The proportion of CMTs was reported at 24.5% (401/1,639) for all canine tumor biopsy cases. Benign and malignant tumors were reported at 14.5% (58/401) and 85.5% (343/401) for all CMT cases, respectively. The mean age of dogs affected by benign CMTs was 9.0 ± 3.0 years, which was significantly lower than for malignant CMTs at 9.9 ± 2.8 years (*P* = 0.0239). According to histopathological classification, benign mixed tumors and simple carcinoma types were highest among benign and malignant CMT cases, respectively. Moreover, female dogs were at significantly higher risk of developing mammary gland tumors (OR = 45.8, 95% CI [3.9–86.0], *P* < 0.0001) than male dogs, as well as older dogs (>8 years) (OR = 1.7, 95% CI [1.2–2.2], *P* = 0.0001) compared to young ones (≤8 years). The proportion of FMTs was 37.1% (36/97) for all feline tumor biopsy cases. Benign and malignant tumors for all FMTs were reported at 16.7% (6/36) and 83.3% (30/36), respectively. According to histopathological classifications, adenoma and simple carcinoma were present in the highest proportion among benign and malignant FMTs, respectively. Female cats were at a significantly higher risk of developing mammary gland tumors than male cats (OR = 25.7, 95% CI [3.9–272.8], *P* < 0.0001).

**Conclusions and clinical importance:**

There was a high proportion of MGT cases compared with other tumor cases reported in a secondary care hospital in Chiang Mai, Thailand from 2012 to 2019, and malignant tumor biopsies have been more frequently observed than benign tumor biopsies in both CMT and FMT cases. The resulting data originating from this study can be an aid for veterinary oncologists in better educating clients and planning treatment and prevention strategies and it can be used as a basis for further experimental studies in the oncology section.

## Introduction

Mammary gland tumors (MGTs) are often found in companion animals worldwide. MGTs development is related to various disease processes including cysts, inflammation, hyperplasia, and benign or malignant mammary tumors (Baker & Lumsden, 1999). Clinical presentations of MGTs are varied. MGTs can be present as single or multiple nodules (Hahn, Bravo & Avenell, 1994). The multiple nodules can be of the same or of different histopathological subtypes (Weijer & Hart, 1983). Clinical behavior ranges from well-circumscribed nodules with stationary growth to large ulcerated nodules that grow rapidly and are fixed to adjacent tissue. However, there may be other indications of malignancy such as those associated with inflammatory carcinoma (Alenza et al., 2000). Canine mammary tumors (CMTs) are the second most common type of tumor found in female dogs (Misdorp, 2002; Reziae et al., 2009). According to medical databases acquired from Texas and Wisconsin, USA, the annual incidence rates of CMTs were reported to be 207 out of 100,000 female dogs and four out of 100,000 male dogs (Lana, Rutteman & Withrow, 2007; Saba et al., 2007), whereas northeastern Italy reported the incidence rate to be 250 out of 100,000 dogs (Vascellari et al., 2016). Sex hormones, such as estrogen and progesterone, tend to play an important role in the early stage of development of mammary tumorigenesis (Rutteman, 1990; Donnay et al., 1996). The late stage of mammary tumorigenesis can be influenced by certain growth factors including epidermal growth factors (EGFs), transforming growth factors (TGFs), and parathyroid hormone-related proteins (Nerurkar et al., 1987; Rutteman, 1990; Okada et al., 1997). Importantly, age and breed are the most significant factors associated with CMT development. Moreover, CMT development has also been reported to be associated with diet, obesity, and any history of carcinogenic exposure (Sonnenschein et al., 1991; Pérez Alenza et al., 1998; Andrade, Figueroa & Bersano, 2010).

Feline mammary tumors (FMTs) are the third most common type of tumor that are known to affect female cats (Dorn et al., 1968). The annual incidence of FMTs has been reported to be 25.4 out of 100,000 cats in the USA (Dorn et al., 1968). A study of FMTs in Italy reported relative frequencies of 16.0% for all tumors found in cats and 25.0% for all tumors found in female cats (Vascellari et al., 2016). FMTs have malignant aggressive phenotypes that are associated with a high metastatic rate (Weijer & Hart, 1983). Previous studies have reported that the risk factors for the occurrence of FMTs can vary depending upon time, location, and population (Hayes, Milne & Mandell, 1981; Overley et al., 2005; Egenvall et al., 2010; Sorenmo et al., 2011). Similar to dogs, ovarian hormones have been strongly implicated in feline mammary tumorigenesis, of which intact female cats have a seven-fold higher risk of undergoing tumorigenesis than spayed cats (Dorn et al., 1968; Overley et al., 2005). These incidences have also been found to be related to aging and breed as specific associated risk factors. Remarkably, the Siamese breed has been identified as a predisposed breed that is more likely to develop FMTs at a younger age than other cat breeds (Hayes, Milne & Mandell, 1981; Overley et al., 2005; Egenvall et al., 2010).

Histopathological examination is a precise diagnostic tool used for MGT diagnosis (Allen, Prasse & Mahaffey, 1986). Poor prognosis has been associated with malignant MGTs due to their high recurrence rate, high metastatic rate, and short survival time (Gilbertson et al., 1983; Rosol et al., 2003; Nguyen et al., 2018). The accumulation of diagnostic recorded data is a key feature in the development of effective prevention and control strategies for animal health management, especially within the field of oncology. However, the availability of data associated with this tumor type in dogs and cats has been limited, particularly in Asian countries. Thus, the objective of this retrospective study was to review the epidemiology of MGT cases, and classification of tumor biopsy results obtained from a secondary care hospital in Chiang Mai, Thailand from 2012 to 2019, and to analyze the factors including sex, age, and breed related to MGT occurrences in dogs and cats.

## Materials & Methods

### Data collection

The retrospective study was conducted by reviewing the report of 1,736 tumor biopsy samples from 1,472 pets (1,639 canine samples from 1,388 dogs and 97 feline samples from 85 cats), which were obtained from the Small Animal Hospital, Faculty of Veterinary Medicine, Chiang Mai University between 2012 and 2019. Among these, 437 samples from 373 dogs and 32 cats were histologically diagnosed as MGTs by multiple pathologists from Veterinary Diagnostic Center, Faculty of Veterinary Medicine, Chiang Mai University in accordance with the standards of WHO histological classification and Histological classification 2010 (Misdorp et al., 1999; Goldschmidt, Pena & Rasotto, 2011). General information pertaining to sex, age, and breed was also recorded.

The study was conducted according to the guidelines of the Institute of Animals for Scientific Purposes Development (IAD), National Research Council of Thailand (NRCT), and approved by the Animal Care and Use Committee (FVM-ACUC), Faculty of Veterinary Medicine, Chiang Mai University (protocol code: S28/2563 and date of approval: October 16, 2020).

### Statistical analyses

The epidemiology and classification of CMTs among all tumor cases were determined using descriptive analysis. In case of coexisting benign and malignant mammary tumors in the same animal, only the malignant mammary tumor was considered for analysis. The ages of the MGTs-affected dogs or cats were reported as mean, median, and standard deviation values. In case of consecutive mammary tumors in the same animal, only the patient age at onset of the first mammary tumor was considered for analysis. The mean age was then compared with those of the benign and malignant tumor groups using an unpaired *T*-test. The risk factors that influenced mammary tumors in dogs and cats were categorized and subsequently analyzed by employing a retrospective case-control study. Specific data attributed to all relevant factors were divided into three categories accordingly; female or male sex, young to middle-aged (≤8 years) or old-aged (>8 years), and purebred or mixed breed. Each factor was separately investigated using Fisher’s exact test. The odds ratio value and 95% confidence interval were computed using the Baptista-Pike method *via* GraphPad Prism 9.1.0 software (GraphPad Software, La Jolla, CA, USA). All values of significance were accepted at *P* < 0.05.

**Figure 1:**
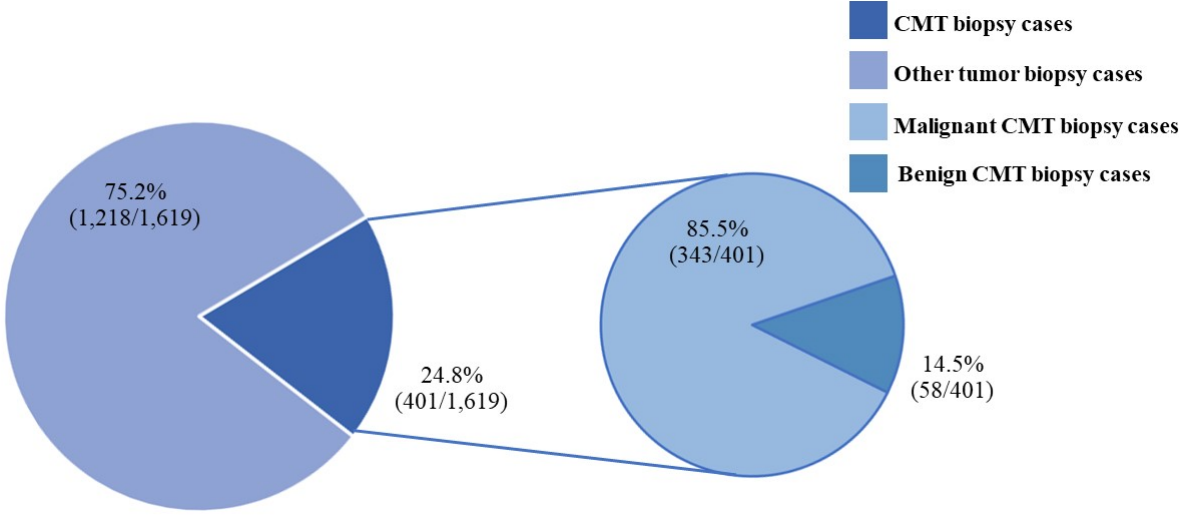
The canine mammary tumor biopsy cases during 2012–2019.

**Figure 2:**
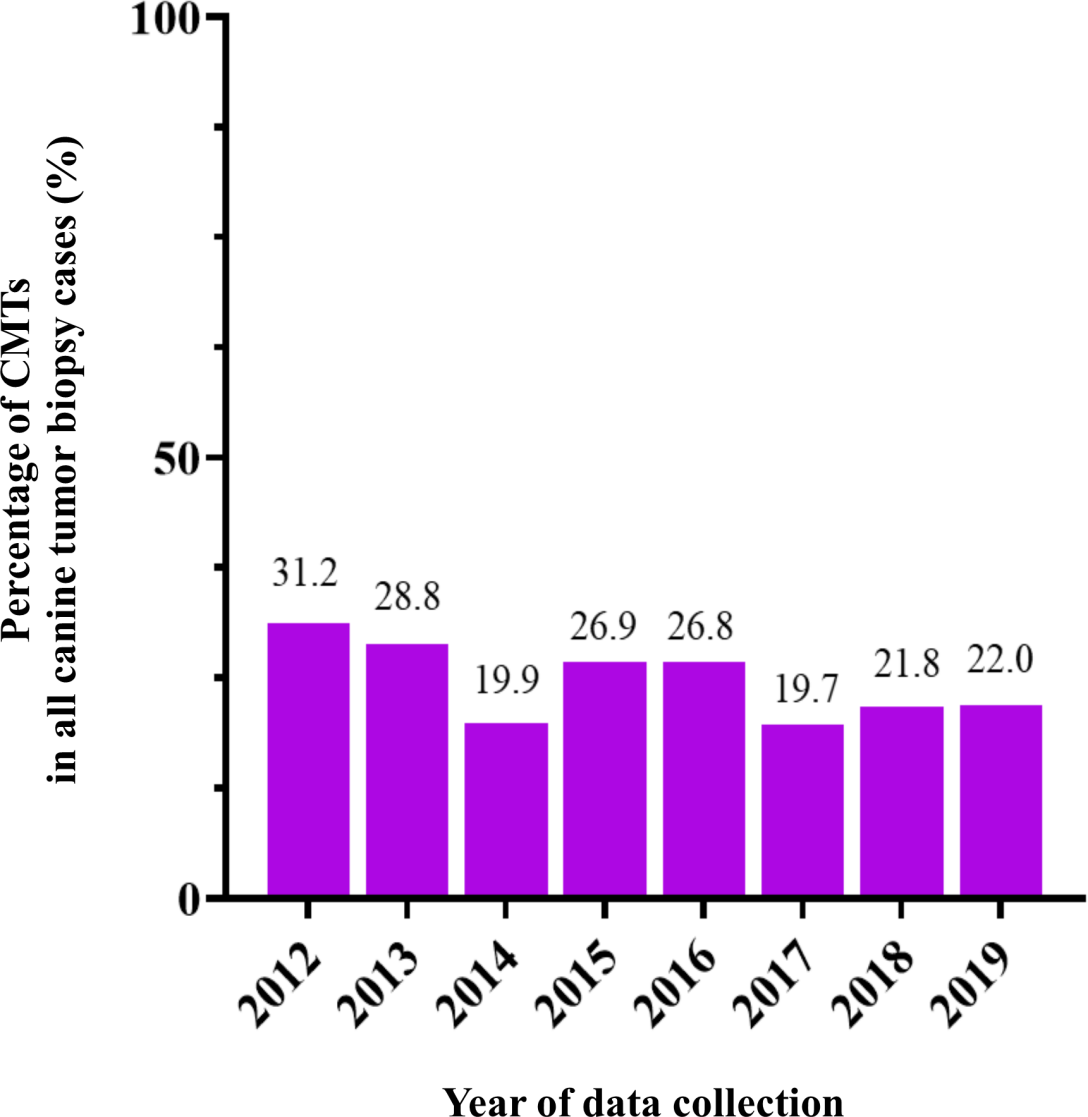
The percentage of canine mammary tumor (CMT) in all tumor biopsy cases in a single year during 2012–2019.

**Figure 3:**
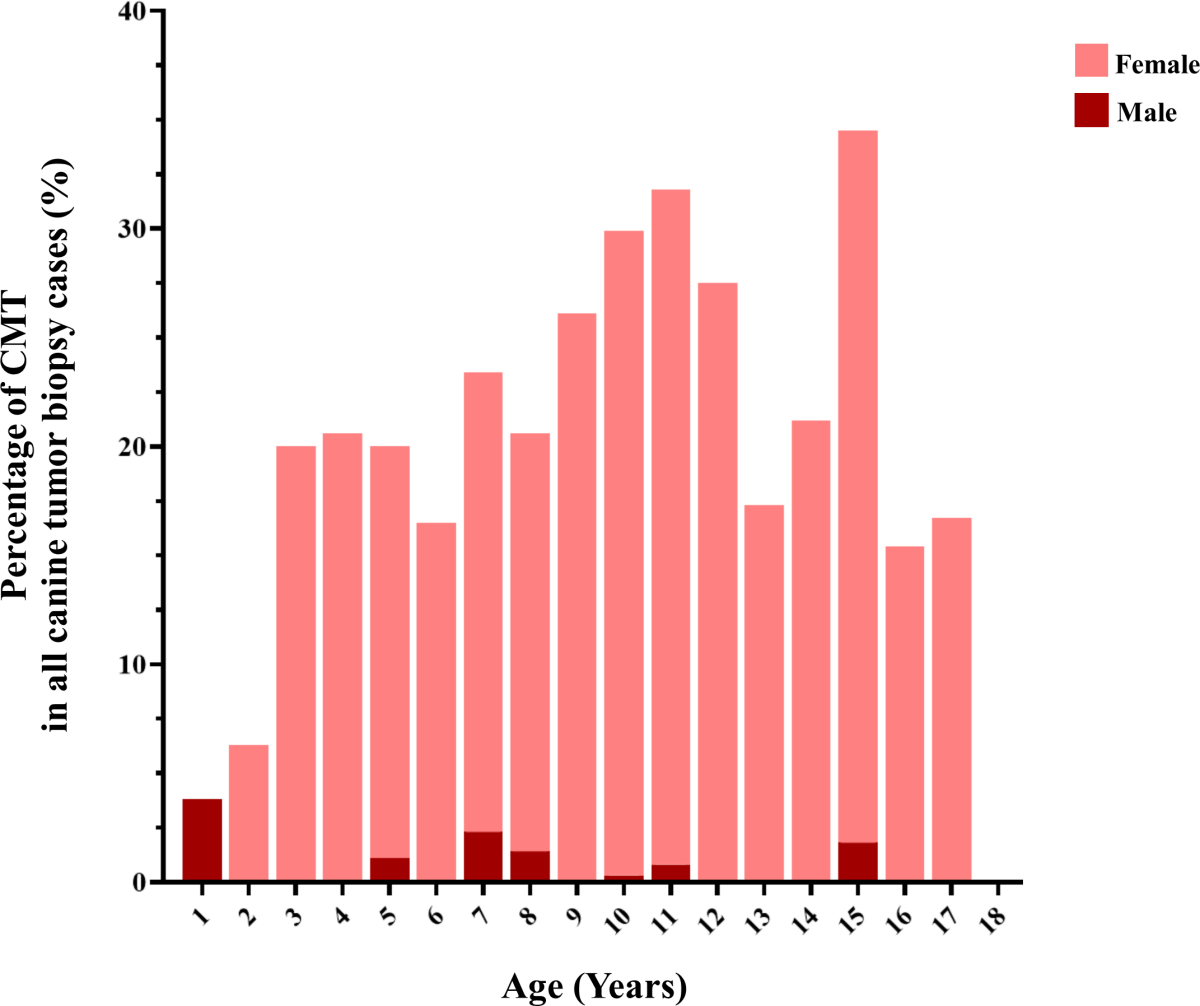
The age of dogs affected by canine mammary tumor (CMT).

## Results

### Epidemiology of CMTs

CMTs were reported at 24.47% (401/1,639) out of all canine tumor biopsy cases during the years from 2012 to 2019, followed by mast cell tumor, lipoma/liposarcoma, and squamous cell carcinoma, which were reported at 10.8% (178/1,639), 6.2% (102/1,639), and 6.0% (99/1,639), respectively (Fig. 1). The CMT biopsy case numbers in a single year tended to decrease from 2012 to 2014, while fewer changes were observed from 2014 to 2019, wherein 21.2–25.4% of all canine tumor biopsy cases were recorded per year (Fig. 2). The proportions of benign and malignant tumors were 14.5% (58/401) and 85.5% (343/401) of all CMT tumor biopsies, respectively. When separated by sex, the proportions of all CMTs in female and male cases were reported at 97.6% (364/373) and 2.4% (9/373), respectively. With regard to benign CMTs, the female and male cases were 96.4% (54/56) and 3.6% (2/56), while among malignant CMTs, the female and male cases were 97.8% (310/317) and 2.2% (7/317), respectively. Furthermore, the data revealed that the age of affected dogs varied between 1–17 years (Fig. 3), while the mean age of cases with CMTs was 9.8 ± 2.9 years (Table 1). Interestingly, the mean age of benign CMT cases, 9.0 ± 3.0 years, was significantly lower than for malignant CMT cases, 9.9 ± 2.8 years (*P* = 0.0239). Regarding breed predispositions, mixed breed dogs had the highest proportion of CMTs (42.6%, 159/343), followed by Poodle (21.7%, 81/343), Shih Tzu (10.5%, 39/343), Golden Retriever (4.8%, 18/343), Cocker Spaniel (3.8%, 14/343), and Labrador Retriever (1.9%, 7/343), respectively (Table 2).

### Classification of CMTs

The histopathological subtype of all CMT cases is shown in Table 3. Among the benign CMTs, benign mixed tumors were observed in the highest proportion at 60.3% (35/58). This was followed by proportions of adenomas and fibroadenoma/fibroma at 32.8% (19/58) and 6.9% (4/58), respectively. Among malignant CMTs, simple carcinoma was observed in the highest proportion at 26.8% (92/343), followed by solid carcinoma and complex carcinoma at proportions of 21.9% (75/343) and 16.6% (57/343), respectively (Table 3).

### Risk factors associated with the occurrence of mammary tumors in dogs

The analysis data presented in Table 4 indicate that female dogs were at a significantly higher risk of developing mammary gland tumors than male dogs (OR = 45.8, 95% CI [23.9–86.0], *P* < 0.0001). Moreover, older dogs of more than 8 years of age were found to be at a significantly higher risk of developing mammary gland tumors than young to middle aged dogs (OR = 1.7, 95% CI [1.3–2.2], *P* = 0.0001). Purebred dogs did not significantly differ in developing mammary gland tumors compared to mixed breed dogs.

### Epidemiology of FMTs

**Figure 4:**
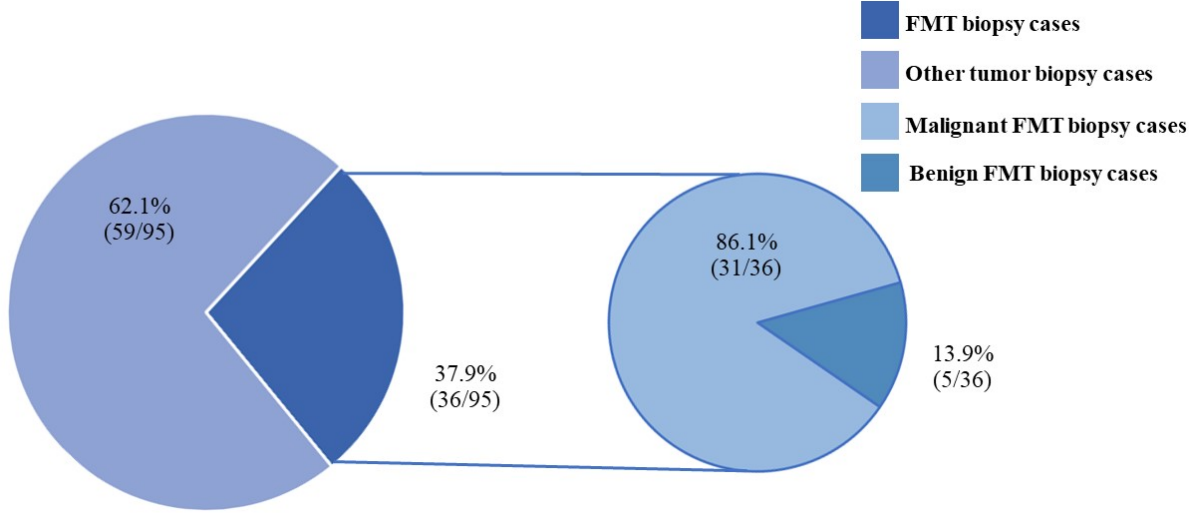
The feline mammary tumor biopsy cases during 2012–2019.

**Figure 5:**
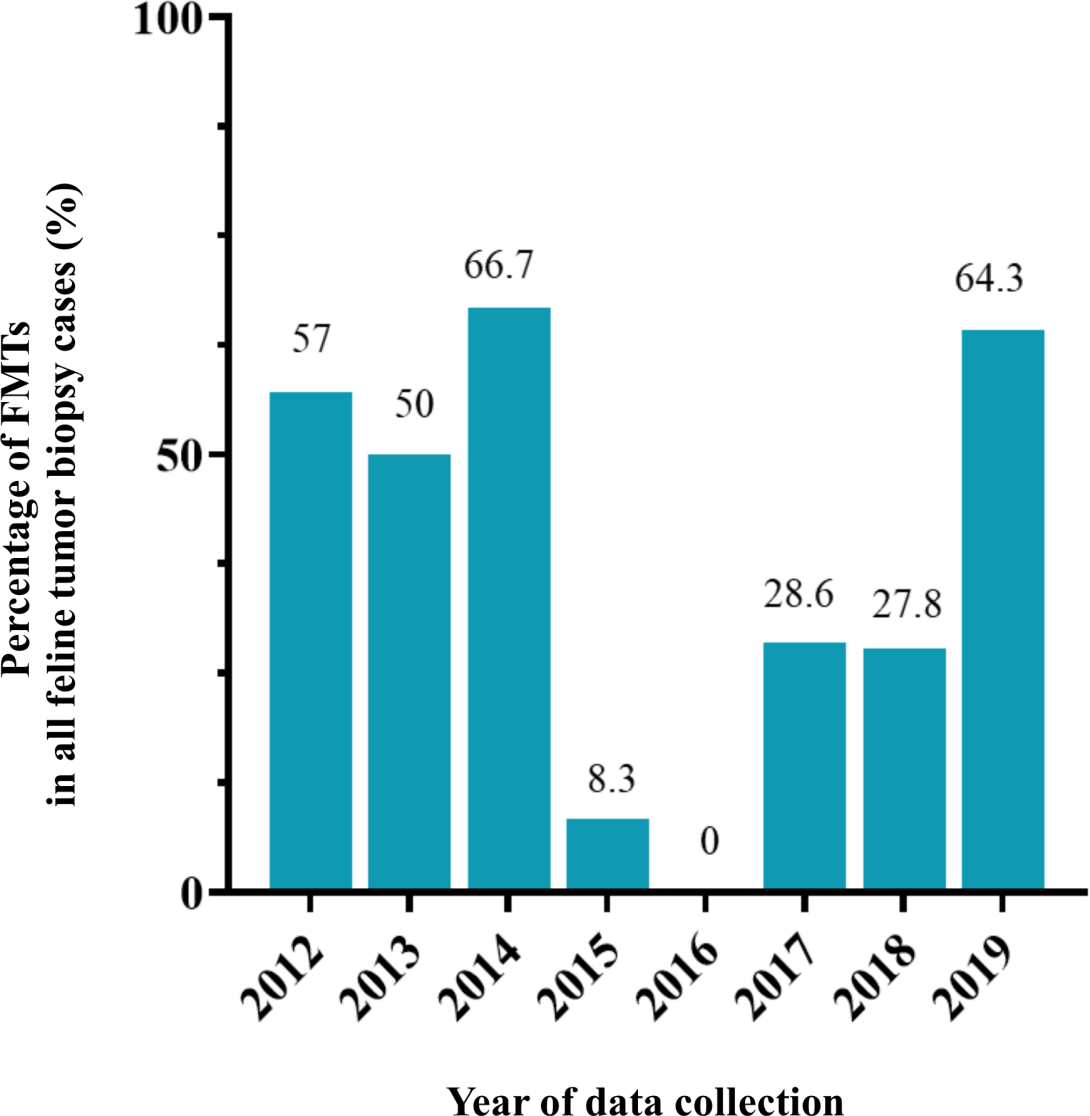
The percentage of feline mammary tumor (FMT) in all tumor biopsy cases in a single year during 2012–2019.

**Figure 6:**
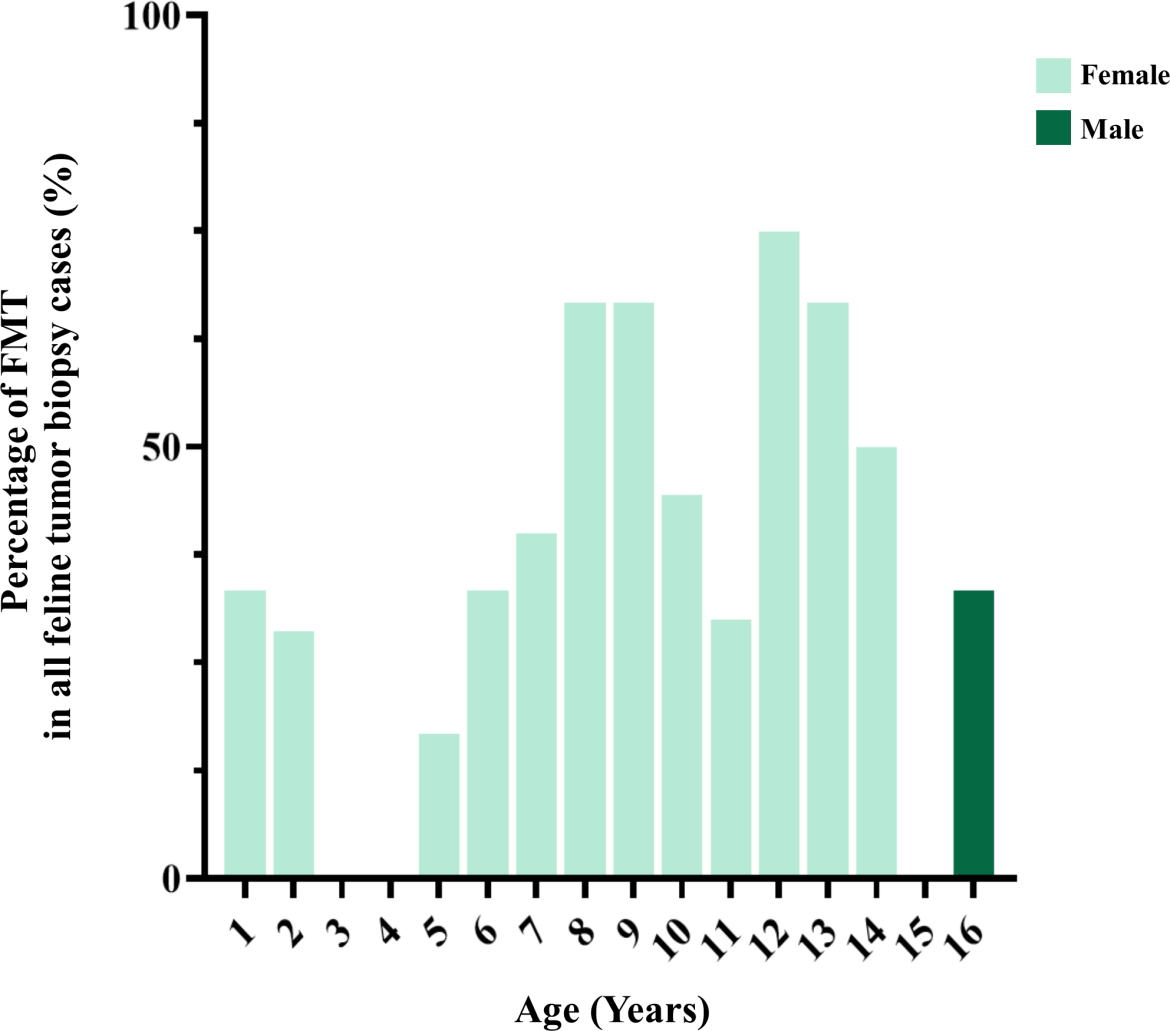
The age of cats affected by feline mammary tumor (FMT).

FMTs were present in 37.1% (36/97) of all feline tumor biopsy cases during the period of 2012 to 2019 followed by Fibroma/Fibrosarcoma, squamous cell carcinoma, and Melanoma/Melanocytoma which were reported at 14.4% (14/97), 10.3% (10/97), and 5.2% (5/97), respectively (Fig. 4). The FMT biopsy case numbers in a single year during the course of this study fluctuated between 0 and 58.3% for all feline tumor biopsy cases per year (Fig. 5). Among all FMTs, the proportions of benign and malignant tumors were 16.7% (6/36) and 83.3% (30/36), respectively, while the female and male cases of FMTs were reported at 96.9% (31/32) and 3.1% (1/32), respectively. Furthermore, the age of affected cats varied between 8 months and 16 years (Fig. 6). The mean age of FMT cases was recorded at 9.0 ± 4.0 years (Table 5). The mean age of benign FMT cases at 6.7 ± 5.9 years was lower than that of malignant FMT cases at 9.4 ± 3.5 years with no significant differences. Additionally, mixed breed cats had the highest proportion of FMTs followed by the Persian breed and an equal number of the Siamese and American Shorthair breeds at 75.0% (24/32), 18.8 (6/32), and 3.1% (1/32), respectively (Table 6).

**Table 1:** The mean age of canine mammary gland tumor (CMT) cases (*n* = 373).

**Type of CMTs**	**Cases** **(n)**	**Minimum** **(Years)**	**Maximum** **(Years)**	**Median** **(Years)**	**Mean ± S.D.** **(Years)**
Benign	56	1.0	15.0	9.0	8.9 ± 3.0^*^
Malignant	315	3.0	17.0	10.0	9.9 ± 2.8
**Total**	**371**	**1.0**	**17.0**	**10.0**	**9.8 ± 2.9**

**Notes.**

Difference superscript asterisk between rows mean significantly different values (*P*-values < 0.05).

**Table 2:** The breed of canine mammary gland tumor (CMT) cases.

**Breed**	**Total cases** **n (%)**	**Non-CMT cases** **n (%)**	**CMT cases** **n (%)**	**Benign CMT cases**		**Malignant CMT cases**	
				**Female** **n (%)**	**Male** **n (%)**	**Female** **n (%)**	**Male** **n (%)**
Alaskan Malamute	1 (0.1)	0 (0.0)	1 (0.3)	0 (0.0)	0 (0.0)	1 (0.3)	0 (0.0)
Alsatian	1 (0.1)	1 (0.1)	0 (0.0)	0 (0.0)	0 (0.0)	0 (0.0)	0 (0.0)
American bully	2 (0.1)	2 (0.2)	0 (0.0)	0 (0.0)	0 (0.0)	0 (0.0)	0 (0.0)
American Pitbull Terrier	11 (0.8)	9 (0.9)	2 (0.5)	0 (0.0)	0 (0.0)	1 (0.3)	1 (14.3)
Bangkaew	25 (1.8)	21 (2.1)	4 (1.1)	0 (0.0)	0 (0.0)	4 (1.3)	0 (0.0)
Basset hound	1 (0.1)	1 (1.0)	0 (0.0)	0 (0.0)	0 (0.0)	04 (0.0)	0 (0.0)
Beagle	15 (1.1)	10 (1.0)	5 (1.3)	1 (1.9)	0 (0.0)	4 (1.3)	0 (0.0)
Boston-terrier	1 (0.1)	1 (0.1)	0 (0.0)	0 (0.0)	0 (0.0)	0 (0.0)	0 (0.0)
Boxer	1 (0.1)	1 (0.1)	0 (0.0)	0 (0.0)	0 (0.0)	0 (0.0)	0 (0.0)
Bull dog	6 (0.4)	5 (0.5)	1 (0.3)	0 (0.0)	0 (0.0)	1 (0.3)	0 (0.0)
Bull terrier	3 (0.2)	3 (0.3)	0 (0.0)	0 (0.0)	0 (0.0)	0 (0.0)	0 (0.0)
Chihuahua	18 (1.3)	11 (1.1)	7 (1.9)	0 (0.0)	0 (0.0)	7 (2.3)	0 (0.0)
Chow Chow	3 (0.2)	2 (0.2)	1 (0.3)	0 (0.0)	0 (0.0)	1 (0.3)	0 (0.0)
Clumber spaniel	1 (0.1)	1 (0.1)	0 (0.0)	0 (0.0)	0 (0.0)	0 (0.0)	0 (0.0)
Cocker Spanial	39 (2.8)	25 (2.5)	14 (3.8)	1 (1.9)	0 (0.0)	13 (4.2)	0 (0.0)
Collie	1 (0.1)	1 (0)	0 (0.0)	0 (0.0)	0 (0.0)	0 (0.0)	0 (0.0)
Dachshund	5 (0.4)	2 (0.2)	3 (0.8)	2 (3.7)	0 (0.0)	1 (2.3)	0 (0.0)
Dalmatian	3 (0.2)	2 (0.2)	1 (0.3)	0 (0.0)	0 (0.0)	1 (0.3)	0 (0.0)
Doberman	1 (0.1)	1 (0.1)	0 (0.0)	0 (0.0)	0 (0.0)	0 (0.0)	0 (0.0)
Dogue de Bordeaux	1 (0.1)	1 (0.1)	0 (0.0)	0 (0.0)	0 (0.0)	0 (0.0)	0 (0.0)
English cocker	3 (0.2)	3 (0.3)	0 (0.0)	0 (0.0)	0 (0.0)	0 (0.0)	0 (0.0)
French bulldog	8 (0.6)	6 (0.6)	2 (0.5)	0 (0.0)	0 (0.0)	2 (0.6)	0 (0.0)
German Shepherd	10 (0.7)	7 (0.7)	3 (0.8)	3 (5.6)	0 (0.0)	0 (0.0)	0 (0.0)
Golden Retriever	112 (8.1)	94 (9.2)	18 (4.8)	4 (7.4)	0 (0.0)	13 (4.2)	1 (14.3)
Jack Russell	1 (0.1)	0 (0.0)	1 (0.3)	1 (1.9)	0 (0.0)	0 (0.0)	0 (0.0)
Labrador Retriever	43 (3.1)	36 (3.5)	7 (1.9)	2 (3.7)	0 (0.0)	5 (1.6)	0 (0.0)
Miniature Pinscher	24 (1.7)	19 (1.9)	5 (1.3)	0 (0.0)	0 (0.0)	5 (1.6)	0 (0.0)
Mixed	645 (46.4)	486 (47.7)	159 (42.6)	19 (35.2)	2 (100.0)	134 (43.2)	4 (57.1)
Pekingese	4 (0.3)	3 (0.3)	1 (0.3)	0 (0.0)	0 (0.0)	1 (0.3)	0 (0.0)
Pomeranian	21 (1.5)	18 (1.8)	3 (0.8)	1 (1.9)	0 (0.0)	2 (0.6)	0 (0.0)
Poodle	177 (12.7)	96 (9.3)	81 (21.7)	13 (24.1)	0 (0.0)	67 (21.6)	1 (14.3)
Pug	14 (1.0)	10 (1.0)	4 (1.1)	1 (1.9)	0 (0.0)	3 (1.0)	0 (0.0)
Rottweiler	11 (0.8)	10 (1.0)	1 (0.3)	0 (0.0)	0 (0.0)	1 (0.3)	0 (0.0)
Saint Bernard	2 (0.1)	2 (0.2)	0 (0.0)	0 (0.0)	0 (0.0)	0 (0.0)	0 (0.0)
Samoyed	1 (0.1)	1 (0.1)	0 (0.0)	0 (0.0)	0 (0.0)	0 (0.0)	0 (0.0)
Schnauzer	3 (0.2)	3 (0.3)	0 (0.0)	0 (0.0)	0 (0.0)	0 (0.0)	0 (0.0)
Shar-pei	1 (0.1)	1 (0.1)	0 (0.0)	0 (0.0)	0 (0.0)	0 (0.0)	0 (0.0)
Shi-tzu	129 (9.3)	90 (8.8)	39 (10.5)	5 (9.3)	0 (0.0)	34 (11.0)	0 (0.0)
Siberian Husky	20 (1.4)	16 (1.6)	4 (1.1)	0 (0.0)	0 (0.0)	4 (1.3)	0 (0.0)
Spitz	6 (0.4)	3 (0.3)	3 (0.8)	1 (1.9)	0 (0.0)	2 (0.6)	0 (0.0)
Thai Ridgeback	10 (0.7)	10 (1.0)	0 (0.0)	0 (0.0)	0 (0.0)	0 (0.0)	0 (0.0)
Yorkshire Terrier	7 (0.5)	4 (0.4)	3 (0.8)	0 (0.0)	0 (0.0)	3 (1.0)	0 (0.0)
	**1,391 (100.0)**	**1,018 (100.0)**	**373 (100.0)**	**54 (100.0)**	**2 (100.0)**	**310 (100.0)**	**7 (100.0)**

**Table 3:** Histopathological classification of canine mammary tumors in a secondary care hospital in Chiang Mai, Thailand during 2012–2019 (*n* = 401).

**Type of CMT** ^1^	**Cases** **n (%)**
**1. Benign**	
**1.1 Adenoma**	**19 (32**.**8)**
**-** Adenoma simple	4 (6.9)
**-** Cystadenoma	6 (10.3)
- Complex adenoma	8 (13.8)
- Basal cell adenoma	1 (1.7)
**1.2 Fibroadenoma/Fibroma**	**4 (6**.**9)**
**1.3 Benign mixed tumor**	**35 (60**.**3)**
**Total**	**58 (100**.**0)**
**2. Malignant**	
**2.1 Carcinoma in situ**	**2 (0**.**6)**
**2.2 Carcinoma: Simple**	**92 (26**.**8)**
- Tubular carcinoma	11 (3.2)
- Tubulo-papillary carcinoma	70 (20.4)
- Cystic-papillary carcinoma	11 (3.2)
**2.3 Carcinoma: Solid**	**75 (21**.**9)**
**2.4 Comedocarcinoma**	**1 (0**.**3)**
**2.5 Carcinoma: Anaplastic**	**46 (13**.**4)**
**2.6 Carcinoma: Complex**	**57 (16**.**6)**
**2.7 Special type of carcinoma**	**10 (2**.**9)**
- Spindle cell carcinoma	1 (0.3)
- Squamous cell carcinoma	6 (1.7)
- Mucinous carcinoma	2 (0.6)
- Lipid-rich carcinoma	1 (0.3)
**2.8 Sarcoma**	**19 (5**.**5)**
- Fibrosarcoma	12 (3.5)
- Osteosarcoma	6 (1.7)
- Myxosarcoma	1 (0.3)
**2.9 Carcinosarcoma**	**41(12**.**0)**
**Total**	**343(100**.**0)**

**Notes.**

1 Histological classification 2010 (Goldschmidt, Pena & Rasotto, 2011).

**Table 4:** The risk factors influencing canine mammary gland tumor (CMT) cases compared with other tumor cases (*N* = 1, 391).

**Factors**	**CMT cases**		**Other tumor cases**		**Odds ratio**	**95% CI**	***P-value***
	**n**	**%**	**n**	**%**			
**1. Sex** (*n* = 1,356)							
Female (*n* = 825)	364	97.6	461	46.9	45.8	23.9–86.0	<0.0001^*^
Male (*n* = 531)	9	2.4	522	53.1	1.0		
**Total**	**373**	**100**.**0**	**983**	**100**.**0**			
**2. Age** (*n* = 1,316)							
≤8 years (*n* = 481)	106	28.4	375	39.8	1.0		
>8 years (*n* = 835)	267	71.6	568	60.2	1.76	1.27–2.2	0.0001^*^
**Total**	**373**	**100**.**0**	**943**	**100**.**0**			
**3. Breed** (*n* = 1,391)							
Purebred (*n* = 753)	214	57.4	539	52.9	1.2	0.9–1.5	0.15
Mixed breed (*n* = 638)	159	42.6	479	47.1	1.0		
**Total**	**373**	**100**.**0**	**1,018**	**100**.**0**			

**Notes.**

* Significant difference was accepted at *P*-value <0.05.

** The retrospective data on sex was not recorded in 35 non-CMT cases.

*** The retrospective data on age was not recorded in 75 non-CMT cases.

### Classification of FMTs

The histopathological results of all FMT cases are presented in Table 7. The benign FMTs were comprised of 66.7% (4/6) simple adenomas followed by the equal of 16.7% (1/6) cystadenoma and 16.7% (1/6) intra-ductal adenoma. Likewise, simple carcinoma displayed the highest proportion among all malignant FMTs followed by solid carcinomas and intraductal papillary carcinomas, whose proportions were 43.3% (13/30), 23.3% (7/30) and 20.0 (6/30), respectively (Table 7).

### Risk factors associated with the occurrence of mammary tumors in cats

The results indicate that female cats were at a significantly higher risk of developing mammary gland tumors than male cats (OR = 25.7, 95% CI [3.9–272.8], *P* < 0.0001). However, age and breed were not significantly different between cats affected with mammary gland tumors and other tumors (Table 8).

## Discussion

The results of a comprehensive survey study will be an important source of information for analyzing neoplastic disease behavior over time. This study has uncovered the classification of epidemiological and histopathological features of mammary gland tumors in dogs and cats in a secondary care hospital in Chiang Mai, Thailand over the period of 2012 to 2019. Furthermore, we have analyzed the risk factors associated with tumors occurring in dogs and cats using case-control study analyses in order to establish relevant scientific information. The resulting data can be used as a basis for further experimental studies.

According to the results, the proportion of CMTs in Thailand is on the top edge of those recorded in previous studies, which reported a proportion within a range of 13.4 to 23.6% for all tumors found in dogs (Dorn et al., 1968; Mulligan, 1975; Grüntzig et al., 2015; Salas et al., 2015). The data would indicate that CMTs are prominent tumors in Thai dogs nowadays. Remarkably, the proportion of malignant tumors in all CMT cases was higher than in earlier studies conducted in Mexico, Norwegian countries, and Italy, where the rates were between 47.5% and 74.0%. These results were similar to those that were reported in Spain and Sri Lanka where the rates were between 88.1% and 87.8% (Moe, 2001; Salas et al., 2015; Rasotto et al., 2017; Ariyarathna et al., 2018; Pastor et al., 2018). These outcomes would have likely been influenced by the time, place, and population of the collected data. Time is an important factor because tumorigenesis is known to be a multi-step process comprised of initiation, promotion, and progression steps. The findings of a number of previous studies have indicated that benign CMTs may arise from small nodules that then develop into large nodules, while benign tumor cells may change to malignant cells (Moulton et al., 1970; Sorenmo et al., 2009; Gedon et al., 2021). Accordingly, late detection by dog owners may have had an effect on the high degree of malignancy case numbers. However, the outcomes of this study should help to make these owners aware of the need to better monitor their dogs. Another explanation for the cause of the high rate of malignant CMTs observed in the present study would be the location of the data collection process and the target population. This study was conducted in a referral hospital, whereas dogs with small nodules, slow growth, and no visible signs of a malignant disease could have been treated by local veterinarians.

**Table 5:** The mean age of feline mammary tumor (FMT) cases (*n* = 32).

**Type of FMT**	**Cases** **(n)**	**Minimum (Years)**	**Maximum** **(Years)**	**Median** **(Years)**	**Mean ± S.D.** **(Years)**
Benign	5	0.7	13.0	7.0	6.7 ± 5.9
Malignant	27	1.0	16.0	10.0	9.4 ± 3.5
**Total**	**32**	**1.0**	**16.0**	**10.0**	**9.0 ± 4.0**

**Notes.**

Difference superscript asterisk between rows mean significantly different values (*P*-values < 0.05).

**Table 6:** The breed of feline mammary gland tumor (FMT) cases.

**Breed**	**Total cases** **n (%)**	**Non-FMT cases** **n (%)**	**FMT cases** **n (%)**	**Benign FMT cases**		**Malignant FMT cases**	
				**Female** **n (%)**	**Male** **n (%)**	**Female** **n (%)**	**Male** **n (%)**
American shorthair	1 (1.2)	0 (0.0)	1 (3.1)	1 (20.0)	0 (0.0)	0 (0.0)	0 (0.0)
Mixed breed	70 (82.4)	46 (86.8)	24 (75.0)	2 (40.0)	0 (0.0)	21 (80.8)	1 (100.0)
Maine Coon	1 (1.2)	1 (1.9)	0 (0.0)	0 (0.0)	0 (0.0)	0 (0.0)	0 (0.0)
Persian	11 (12.9)	5 (9.4)	6 (18.8)	2 (40.0)	0 (0.0)	4 (15.4)	0 (0.0)
Siamese	2 (2.4)	1 (1.9)	1 (3.1)	0 (0.0)	0 (0.0)	1 (3.8)	0 (0.0)
	**85** **(100.0)**	**53** **(100.0)**	**32** **(100.0)**	**5** **(100.0)**	**0** **(0.0)**	**26** **(100.0)**	**1** **(100.0)**

**Table 7:** Histopathological classification of feline mammary tumors (FMTs) in a secondary care hospital in Chiang Mai, Thailand during 2012–2019 (*n* = 36).

**Type of FMT** ^1^	**Cases** **n (%)**
**1. Benign**	
**1.1 Adenoma**	**6 (100**.**0)**
**-** Adenoma simple	4 (66.7)
**-** Cystadenoma	1 (16.7)
- Intraductal adenoma	1 (16.7)
**Total**	**6 (100**.**0)**
**2. Malignant**	
**2.1 Carcinoma: Simple**	**13 (43**.**3)**
- Tubular carcinoma	6 (20.0)
- Tubulo-papillary carcinoma	5 (16.7)
- Cystic-papillary carcinoma	1 (3.3)
- Cribriform carcinoma	1 (3.3)
**2.2 Carcinoma: Solid**	**7 (23**.**3)**
**2.3 Comedocarcinoma**	**1 (3**.**3)**
**2.4 Ductal carcinoma**	**1 (3**.**3)**
**2.5 Intraductal papillary carcinoma**	**6 (20**.**0)**
**2.6 Sarcoma**	**1 (3**.**3)**
- Osteosarcoma	1 (3.3)
**2.7 Carcinosarcoma**	**1 (3**.**3)**
**Total**	**30 (100**.**0)**

**Notes.**

1 Adapt from Histological classification 1999 (Misdorp et al., 1999)

**Table 8:** The risk factors influencing feline mammary gland tumor (FMT) cases compared with other tumors cases (*N* = 85).

**Factors**	**FMT cases**		**Other tumor cases**		**Odds ratio**	**95% CI**	***P-value***
	**n**	**%**	**n**	**%**			
**1. Sex (*n* = 85)**							
Female (*n* = 60)	31	96.9	29	54.7		25.7	3.9–272.8	<0.0001^*^
Male (*n* = 25)	1	3.1	24	45.3		1.0		
**Total**	**32**	**100**.**0**	**53**	**100**.**0**				
**2. Age (*n* = 78)**							
≤8 years (*n* = 41)	13	40.6	28	60.9		1.0		
>8 years (*n* = 37)	19	59.4	18	39.1		2.3	0.9–5.8	0.1072
**Total**	**32**	**100**.**0**	**46**	**100**.**0**				
**3. Breed (*n* = 85)**							
Purebred (*n* = 15)	8	25	7	13.6		2.2	0.7–6.3	0.2401
Mixed breed (*n* = 70)	24	75	46	86.4		1.0		
**Total**	**32**	**100.0**	**53**	**100**.**0**				

**Notes.**

* Significant difference was accepted at *P*-value < 0.05.

** The retrospective data on age was not recorded in 7 non-FMT cases.

The histological classification results observed in the present study agree with those of previous reports, wherein mixed tumors were associated with the highest number of benign CMTs followed by adenomas and fibroadenomas (Salas et al., 2015; Rasotto et al., 2017). The normal mammary glands of dogs are composed of luminal epithelial cells, myoepithelial cells, and interstitial connective tissues; thus, benign tumors that originate from a mix of these cell types can commonly be present (Misdorp et al., 1999; Goldschmidt, Pena & Rasotto, 2011). However, the literal cause of the occurrence of tumorigenesis in mixed tumors is not known. Accordingly, there are four theories of histogenesis that have been proposed: (1) the collision theory, where two independent tumors are located adjacent to each other; (2) the combination theory, where stem or multipotent progenitor cells are the origins and the tumors are monoclonal; (3) the conversion or metaplastic theory, where neoplastic myoepithelial/basal cells change their phenotype by metaplasia or trans-differentiation, and (4) the cancer stem cells (CSCs) origin theory, where CSCs differentiate into neoplastic luminal and myoepithelial cells and further differentiate into neoplastic chondrocytes and osteoblasts following certain factor stimulations (Hellmén, 2005; Sánchez-Céspedes et al., 2016; Michishita, 2020). Although benign CMTs are considered rare in male dogs according to a previously referenced study, only one case of adenoma was reported in the present study (Saba et al., 2007).

In addition, the histological classification results of malignant CMT types agree with those that were reported in earlier studies, for which simple carcinomas were associated with the highest number of all malignant CMTs (Ezerskyte et al., 2011; Tavasoly et al., 2013; Rasotto et al., 2017). In 2011, Ežerskytė et al. (2011) reported that the most common malignant CMT types were simple carcinoma, complex carcinoma, and carcinosarcoma, for which the proportions were 46.0%, 27.0%, and 13.0%, respectively. Later, Tavasoly et al. (2013) reported that the most common malignant CMT types were simple carcinoma followed by complex carcinoma, sarcoma, carcinoma arising from benign tumors, and special types of carcinomas, for which the proportions were 56.8%, 13.5%, 13.5%, 10.8%, and 5.4%, respectively. Additionally, Rasotto et al. (2017) reported that simple carcinomas were associated with the highest proportion followed by complex carcinoma, solid carcinoma, carcinosarcoma, malignant myoepithelioma, anaplastic carcinoma, and others. The rates of proportion of those studies and our study were difficult to compare due to the fact that different references in classifications could have led to differences in some of the relevant details. However, the ultimate goal of this histologic classification survey of CMTs is to accurately predict the biological behavior of tumors and to contribute to the existing databases of veterinarian oncology in order to achieve the most accurate prognosis possible.

In the present study, the data pertaining to the risk factors related to the occurrence of CMTs consisted of sex and age, which had also been applied in many previous studies (Schneider, 1970; Dorn et al., 1968; Hellmén et al., 1993; Salas et al., 2015; Vascellari et al., 2016; Burrai et al., 2020). Likewise, our descriptive results on sex and age support the previously published findings. Those findings indicate that female dogs had higher numbers of CMTs than male dogs, while their mean age was within a range of 9 to 11 years (Schneider, 1970; Hellmén et al., 1993; Alenza et al., 2000; Zatloukal et al., 2005). Moreover, the mean age of benign CMT-affected dogs tended to be lower than that of malignant CMT-affected dogs (Zatloukal et al., 2005).

Hormonal factors play a major role in the early stage of mammary tumorigenesis in dogs, especially sex steroid hormones (Rutteman, 1990; Donnay et al., 1996). Among female dogs going through puberty, mammary development is initiated by the release of estrogen from the ovaries. Mammary growth involves the lengthening and branching of the ductal system of the lobes and the developing alveoli and secretory units that are supported by a rise in progesterone during pregnancy. Accordingly, the alveolar cell changes to a secretory alveolar cell under the influence of prolactin. Subsequently, mammary gland regression is replaced after this cycle is finished (Sorenmo et al., 2011). Even though the estrous cycle occurs without pregnancy, both development and regression are related to those occurrences with the exception of the changing of a secretory cell (Chandra, Cline & Adler, 2010). Normal mammary gland development and regression cycles are also known to be related to mitosis (Rasotto et al., 2014). Asymmetric cell division may occur and can be the cause of tumorigenesis (Chhabra & Booth, 2021). According to the risk of asymmetric cell division during mammary gland development and the regression cycle, female or middle-aged to old-aged dogs may experience a higher risk of CMT occurrence than male or young dogs. This paradigm was supported by the findings of a previous study, which indicated that dogs that had stopped their estrous cycles through early spaying exhibited a lower risk of developing CMTs than other dogs (Schneider, Dorn & Taylor, 1969; Beaudu-Lange et al., 2021).

Fewer epidemiologic studies have been conducted that focus on incidences of MGTs in cats when compared with dogs. The degree of proportion of FMTs in this study in overall was higher than in previously reported studies at approximately 17.0% of all feline tumors (Misdorp & Weijer, 1980; Hayes & Mooney, 1985; Vascellari et al., 2009). The high frequency of benign and malignant FMT occurrence identified in the present study was similar to that of earlier reports within a range of 80–96% (Hayes, Milne & Mandell, 1981; Misdorp, Romijn & Hart, 1991; Overley et al., 2005). However, the number of FMT biopsy cases can fluctuate over a single year of collection, especially in 2016 when the total number of FMT is zero. This situation might be explained in terms of the number of cases. There was a lower number of oncological cases from primary care hospitals in 2016 than in other years in this study. Likewise, this situation can occur in the statistical study of the low number of populations. Therefore, this should be noted for comparison with other studies in proportion.

According to histopathological classifications, this study revealed that incidences of simple adenoma, cystadenoma and intraductal adenoma (duct papilloma) were mainly observed among benign FMT cases. This outcome was in contrast with the findings of a previous study, wherein these incidences were rarely reported (Hampe & Misdorp, 1974; Caliari et al., 2014). In contrast, the histopathological features of malignant FMTs in our study agreed with those of previous reports, wherein the majority of malignant FMTs were simple carcinoma cases followed by various other types of carcinoma cases (Castagnaro et al., 1998; Caliari et al., 2014; Chocteau et al., 2019). Castagnaro et al. (1998) reported that tubular carcinomas were the most frequently represented tumor type at 47.3% followed by solid carcinomas and papillary carcinomas at 36.4% and 16.4%, respectively (Castagnaro et al., 1998). Furthermore, Caliari et al. (2014) reported that tubular carcinoma (32.0%) and tubulopapillary carcinoma (26.5%) were most common. These reported cases were followed by cases of comedocarcinoma (15.0%), ductal carcinoma (9.0%), solid carcinoma (6.0%), squamous cell carcinoma (4.5%), cribriform carcinoma (3.0%), intraductal papillary carcinoma (3.0%), and carcinoma-in-situ (1.5%) (Caliari et al., 2014). Additionally, the predominant types of malignant FMTs were cribriform carcinoma (46.6%), solid carcinoma (17.2%), tubulopapillary carcinoma (11.4%), mucinous carcinoma (9.4%), tubular carcinoma (7.1%), and papillary carcinoma (5.6%) (Chocteau et al., 2019). In contrast to the findings of previous studies, the outcomes of this study indicate that osteosarcomas and carcinosarcomas were found sporadically among animals diagnosed with malignant FMTs.

The analysis results support the contention that female cats represent a predisposition risk factor for the occurrence of FMT occurrence. This determination was similar to the findings of an earlier study, which reported that female cats were at a higher risk of FMT occurrence than males by 23 times (Vascellari et al., 2009). This outcome could be explained by the presence of a hormonal factor-related FMT. Notably, sex steroid hormones may be implicated in FMT development. This paradigm is supported by the outcomes of an earlier study, which reported that intact cats are more likely to develop MGTs than spayed cats (Overley et al., 2005). Moreover, cats receiving a regular dose of synthetic analog progesterone experienced an increased risk of MGT occurrence than cats who did not receive it (Misdrop, 1988).

Interestingly, the mean age of cats affected by MGTs in Thailand was lower than in other studies and was between 11.1 and 12.8 years (Ito et al., 1996; Overley et al., 2005; Skorupski et al., 2005; Petrucci et al., 2020). Previous studies have indicated that the FMTs that occurred at a younger age may be associated with certain genetic relations. Siamese cats, one of several varieties of native Thai cats that have become popular breeds in Europe and North America in the 19th century, are more likely to develop mammary tumors and at a younger age (9.0 years) than other cat breeds (14.0 years) (Hayes, Milne & Mandell, 1981; Egenvall et al., 2010). Nowadays, native Thai purebred cats are in fewer numbers in Thailand due to the free-living culture of husbandry and the natural mating behaviors of cats, which has led to the presence of a high number of mixed breed cats throughout the country.

However, our study lacks certain other factors, such as spaying status and the use of synthetic hormones which might relate to prognosis in analysis results. In addition, the analyses for assessing the correlations between the signalments of patients (such as the spay status), histopathological features of mammary tumor tissues (such as the grading, primary or metastasized site, immune cell infiltration), and mammary tumor subtypes should be highlighted in further investigations when examining the epidemiology occurrence of CMT in dogs and FMTs in cats in a secondary care hospital.

## Conclusions

This study highlighted a high number of MGTs in dogs and cats in a secondary care hospital in Chiang Mai, Thailand. According to histopathological features, malignant tumors are more frequently observed than benign tumors in both CMT and FMT cases. The findings of our study support the determination that female dogs and cats were mainly affected by MGTs. Moreover, purebred or old aged (>8 years) dogs have a significantly increased risk of CMT occurrence when compared with mixed breed or young to middle-aged (≤8 years) dogs. In contrast, in this study, breed and age were not found to be related to the occurrence of FMTs.

## Supplemental Information

10.7717/peerj.17077/supp-1 Supplemental Information 1 Analyzed data

10.7717/peerj.17077/supp-2 Supplemental Information 2 Raw data from SAH

10.7717/peerj.17077/supp-3 Supplemental Information 3 Raw data

## Additional Information and Declarations

### Data Availability

The following information was supplied regarding data availability: The raw measurements are available in the Supplementary Files.

## References

[ref-1] Alenza MD, Pena L, Del Castillo N, Niet AI (2000). Factors influencing the incidence and prognosis of canine mammary tumours. Journal of Small Animal Practice.

[ref-2] Allen SW, Prasse KW, Mahaffey EA (1986). Cytologic differentiation of benign from malignant canine mammary tumors. Veterinary Pathology.

[ref-3] Andrade F, Figueroa F, Bersano P (2010). Malignant mammary tumor in female dogs: environmental contaminants. Diagnostic Pathology.

[ref-4] Ariyarathna H, de Silva N, Aberdein D, Kodikara D (2018). A clinicopathological diversity of canine mammary gland tumors in Sri Lanka: a one-year survey on cases presented to two veterinary practices. Veterinary Sciences.

[ref-5] Baker RH, Lumsden JH (1999). Color atlas of cytology of the dog and cat.

[ref-6] Beaudu-Lange C, Larrat S, Lange E, Lecoq K, Nguyen F (2021). Prevalence of reproductive disorders including mammary tumors and associated mortality in female dogs. Veterinary Sciences.

[ref-7] Burrai GP, Gabrieli A, Moccia V, Zappulli V, Porcellato I, Brache C (2020). A statistical analysis of risk factors and biological behavior in canine mammary tumors: a multicenter study. Animals.

[ref-8] Caliari D, Zappulli V, Rasotto R, Cardazzo B, Frassineti F, Goldschmidt MH, Castagnaro M (2014). Triple-negative vimentin-positive heterogeneous feline mammary carcinomas as a potential comparative model for breast cancer. BMC Veterinary Research.

[ref-9] Castagnaro M, Bozzetta E, de Maria R, Biolatti B, Caramelli M (1998). Tumour grading and the one-year post-surgical prognosis in feline mammary carcinomas. Journal of Comparative Pathology.

[ref-10] Chandra SA, Cline JM, Adler RR (2010). Cyclic morphological changes in the Beagle mammary gland. Toxicologic Pathology.

[ref-11] Chhabra SN, Booth BW (2021). Asymmetric cell division of mammary stem cells. Cell Division.

[ref-12] Chocteau F, Boulay M, Besnard F, Valeau G, Loussouarn D, Nguyen F (2019). Proposal for a histological staging system of mammary carcinomas in dogs and cats. Par 2: feline mammary carcinomas. Frontier in Veterinary Science.

[ref-13] Donnay I, Devleeschower N, Wouters-Ballman P, Leclerq G, Verstegen J (1996). Relationship between receptors for epidermal growth factor and steroid hormones in normal, dysplastic and neoplastic canine mammary tissues. Research in Veterinary Science.

[ref-14] Dorn CR, Taylor DON, Schneider R, Hibbard HH, Klauber MR (1968). Survey of animal neoplasms in Alameda and Contra Costa Counties, California. II. Cancer morbidity in dogs and cats from Alameda County. Journal of the National Cancer Institute.

[ref-15] Egenvall A, Bonnett BN, Haggstrom J, Ström Holst B, Möller L, Nødtvedt A (2010). Morbidity of insured Swedish cats during 1999–2006 by age, breed, sex, and diagnosis. Journal of Feline Medicine and Surgery.

[ref-16] Ežerskytė A, Zamokas G, Geigonis A, Juodžiukynienė N (2011). The retrospective analysis of mammary tumors in dogs. Verinarija IR Zootechnika.

[ref-17] Gedon J, Wehrend A, Failing K, Kessler M (2021). Canine mammary tumours: size matters-a progression from low to highly malignant subtypes. Veterinary and Comparative Oncology.

[ref-18] Gilbertson S, Kurzman I, Zachrau R, Hurvitz A, Black M (1983). Canine mammary epithelial neoplasms: biologic implications of morphologic characteristics assessed in 232 dogs. Veterinary Pathology.

[ref-19] Goldschmidt M, Peña L, Rasotto R (2011). Classification and grading of canine mammary tumors. Veterinary Pathology.

[ref-20] Grüntizig K, Graf M, Hässig M, Welle M, Meier D, Lott G, Erni D, Schenker NS, Guscetti F, Boo G, Axhausen K, Fabrikant S, Folkers G, Pospischil A (2015). The swiss canine cancer registry: a retrospective study on the occurrence of tumours in dogs in Switzerland from 1955 to 2008. Journal of Comparative Pathology.

[ref-21] Hahn KA, Bravo L, Avenell JS (1994). Feline breast carcinoma as a pathologic and therapeutic model for human breast cancer. In Vivo.

[ref-22] Hampe JF, Misdorp W (1974). Tumours and dysplasias of the mammary gland. Bulletin of the World Health Organization.

[ref-23] Hayes AA, Mooney S (1985). Feline mammary tumors. The veterinary clinics of North America. Small Animal Practice.

[ref-24] Hayes HM, Milne KL, Mandell CP (1981). Epidemiological features of feline mammary carcinoma. The Veterinary Record.

[ref-25] Hellmén E (2005). Complex mammary tumours in the female dog: a review. The Journal of Dairy Research.

[ref-26] Hellmén E, Bergström R, Holmberg L, Spångberg IB, Hansson K, Lindgren A (1993). Prognostic factors in canine mammary tumors: a multivariate study of 202 consecutive cases. Veterinary. Pathology.

[ref-27] Ito T, Kadosawa T, Moshisuki M, Matsunaga S, Nishimura R, Sasaki N (1996). Prognosis of malignant mammary tumor of 53 cats. Journal of Veterinary Medical Science.

[ref-28] Lana SE, Rutteman GR, Withrow SJ, Withrow SJ, Vail DM (2007). Tumors of the mammary gland. Small animal clinical oncology.

[ref-29] Michishita M (2020). Understanding of tumourigenesis in canine mammary tumours based on cancer stem cell research. The Veterinary Journal.

[ref-30] Misdrop W (1988). Canine mammary tumours: protective effect of late ovariectomy and stimulating effect of progestins. Veterinary Quarterly.

[ref-31] Misdorp W, Meuten DJ (2002). Tumors of the mammary gland. Tumors in domestic animals.

[ref-32] Misdorp W, Else RW, Hellmen E, Lipscomb TP (1999). Histologic classification of mammary tumors of the dog and the cat.

[ref-33] Misdorp W, Romijn A, Hart AA (1991). Feline mammary tumors: a case-control study of hormonal factors.

[ref-34] Misdorp W, Weijer K (1980). Animal model of human disease: breast cancer. The American Journal of Pathology.

[ref-35] Moe L (2001). Population-based incidence of mammary tumours in some dog breeds. Journal of Reproduction and Fertility. Supplement.

[ref-36] Moulton JE, Taylor DON, Dorn CR, Andersen AC (1970). Canine mammary tumors. Pathologia Veterinaria.

[ref-37] Mulligan RM (1975). Mammary cancer in the dog; a study of 120 cases. American Journal of Veterinary Research.

[ref-38] Nerurkar VR, Seshadri R, Mulherkar R, Ishwad CS, Lalitha VS, Naik SN (1987). Receptors for epidermal growth factor and estradiol in canine mammary tumors. International Journal of Cancer.

[ref-39] Nguyen F, Peña L, Ibisch C, Loussouarn D, Gama A, Rieder N, Belousov A, Campone M, Abadie J (2018). Canine invasive mammary carcinomas as models of human breast cancer. Part 1: natural history and prognostic factors. Breast Cancer Research and Treatment.

[ref-40] Okada H, Nishijima Y, Yoshino A, Capen CC, Rosol TJ (1997). Immunohistochemical localization of parathyroid hormone-related protein in canine mammary tumors. Veterinary Pathology.

[ref-41] Overley B, Shofer FS, Goldschmidt MH, Sherer D, Sorenmo KU (2005). Association between ovarihysterectomy and feline mammary carcinoma. Journal of Veterinary Internal Medicine.

[ref-42] Pastor N, Caballé, Santella M, Ezquerra LJ, Tarazona R, Duran E (2018). Epidemiological study of canine mammary tumors: age, breed, size and malignancy. Australian Journal of Veterinary Science.

[ref-43] Pérez Alenza D, Rutteman GR, Peña L, Beynen AC, Cuesta P (1998). Relation between habitual diet and canine mammary tumors in a case-control study. Journal of Veterinary Internal Medicine.

[ref-44] Petrucci G, Henriques J, Gregório H, Vicente G, Prada J, Pires I, Lobo L, Medeiros R, Queiroga F (2020). Metastatic feline mammary cancer: prognostic factors, outcome and comparison of different treatment modalities—a retrospective multicentre study. Journal of Feline Medicine and Surgery.

[ref-45] Rasotto R, Berlato D, Goldschmidt MH, Zappulli V (2017). Prognostic significance of canine mammary tumor histologic subtypes: an observational cohort study of 229 cases. Veterinary Pathology.

[ref-46] Rasotto R, Goldschmidt H, Castagnaro M, Carnier P, Caliari D, Zappulli V (2014). The dog as a natural animal model for study of the mammary myoepithelial basal cell lineage and its role in mammary carcinogenesis. Journal of Comparativet Pathology.

[ref-47] Reziae A, Tavasoli A, Bahonar A, Mehrazma M (2009). Grading in canine mammary gland carcinoma. Journal of Biological Sciences.

[ref-48] Rosol TJ, Tannehill-Gregg SH, LeRoy BE, Mandl S, Contag CH (2003). Animal models of bone metastasis. Cancer.

[ref-49] Rutteman GR (1990). Hormones and mammary tumour disease in the female dog: an update. In Vivo.

[ref-50] Saba CF, Rogers KS, Newman SJ, Mauldin GE, Vail DM (2007). Mammary gland tumors in male dogs. Journal of Veterinary Internal Medicine.

[ref-51] Salas Y, Márquez A, Diaz D, Romero L (2015). Epidemiological study of mammary tumors in female dogs diagnosed during the period 2002–2012: a growing animal health problem. PLOS ONE.

[ref-52] Sánchez-Céspedes R, Millán Y, Guil-Luna S, Reymundo C, de Los Monteros AE, de Las Mulas JM (2016). Myoepithelial cells in canine mammary tumours. Veterinary Journal.

[ref-53] Schneider R (1970). Comparison of age, sex, and incidence rates in human and canine breast cancer. Cancer.

[ref-54] Schneider R, Dorn CR, Taylor DO (1969). Factor influencing canine mammary cancer developments and post-surgical survival. Journal of the National Cancer Institute.

[ref-55] Skorupski KA, Overley B, Shofer FS, Goldschmidt MH, Miller CA, Sørenmo KU (2005). Clinical characteristics of mammary carcinoma in male cats. Journal of Veterinary Internal Medicine.

[ref-56] Sonnenschein EG, Glickman LT, Goldschmidt MH, McKee LJ (1991). Body conformation, diet, and risk of breast cancer in pet dogs: a case-control study. American Journal of Epidemiology.

[ref-57] Sorenmo KU, Kristiansen VM, Cofone MA, Shofer FS, Breen AM, Langeland M, Mongil CM, Grondahl AM, Teige J, Goldschmidt MH (2009). Canine mammary gland tumours; a histological continuum from benign to malignant; clinical and histopathological evidence. Veterinary and Comparative Oncology.

[ref-58] Sorenmo KU, Rasotto R, Zappulli V, Goldschmidt MH (2011). Development, anatomy, histology, lymphatic drainage, clinical features, and cell differentiation markers of canine mammary gland neoplasms. Veterinary Pathology.

[ref-59] Tavasoly A, Golshahi H, Rezaie A, Farhadi M (2013). Classification and grading of canine malignant mammary tumors. Veterinary Research Forum.

[ref-60] Vascellari M, Baioni E, Ru G, Carminato A, Mutinelli F (2009). Animal tumour registry of two provinces in northern Italy: incidence of spontaneous tumours in dogs and cats. BMC Veterinary Research.

[ref-61] Vascellari P, Capello K, Carminato A, Zanardello C, Baioni E, Mutinelli F (2016). Incidence of mammary tumors in the canine population living in the Veneto region (Northeastern Italy): risk factors and similarities to human breast cancer. Preventive Veterinary Medicine.

[ref-62] Weijer K, Hart AA (1983). Prognostic factors in feline mammary carcinoma. Journal of the National Cancer Institute.

[ref-63] Zatloukal J, Lorenzová J, Tichý, Nečas A, Kecova H, Kohout P (2005). Breed and age as risk factors for canine mammary tumours. Acta Veterinaria Brno.

